# Response of photosynthesis, population physiological indexes, and yield of cotton in dry areas to the new technology of “dry sowing and wet emergence”

**DOI:** 10.3389/fpls.2024.1487832

**Published:** 2024-10-17

**Authors:** Yu Ding, Jianqin Ma, Jianghui Zhang, Yungang Bai, Bifeng Cui, Xiuping Hao, Guangtao Fu, Ming Zheng, Bangxin Ding

**Affiliations:** ^1^ College of Water Resources, North China University of Water Resources and Electric Power, Zhengzhou, China; ^2^ Xinjiang Institute of Water Resources and Hydropower Research, Drought and Water Hazard Defence Institute, Urumqi, China; ^3^ Centre for Water Systems, School of Environmental Science and Economics, University of Exeter, Exeter, England; ^4^ Key Laboratory of Agricultural Soil and Water Engineering in Arid and Semiarid Areas, Ministry of Education, Northwest A&F University, Xianyang, China

**Keywords:** arid region, photosynthesis, population physiological index, quality, water management, yield

## Abstract

**Introduction:**

In arid areas, exploring new "dry sowing wet emergence (DSWE)" water-saving irrigation techniques may become one of the most important ways to reduce agricultural irrigation water use and improve economic efficiency.

**Methods:**

The study was conducted in a two-year field trial in 2021 and 2022, setting up three seedling emergence rates (W1: 6 mm, W2: 10.5 mm, W3: 15 mm) and two drip frequencies (D1: 2 times, D2: 4 times) for a total of six irrigation combinations.

**Results and discussion:**

The results indicate that under the "DSWE" irrigation pattern, in contrast to the low frequency treatment, the photosynthetic efficiency of cotton leaves in the high-frequency treatment is significantly higher. The stomatal conductance of cotton leaves has increased by 6.67% within two years, and the net photosynthetic rate has risen by 12.22%. Compared with the CK treatment, there is no remarkable difference in the photosynthetic indicators of the W3D2 treatment, while the net photosynthetic rate has increased by 1.68%. The population physiological indicators of each treatment group exhibit a trend of initially increasing and then decreasing as the growth period prolongs. The differences in the group population physiological indicators of cotton at the seedling stage among different seedling water treatments are relatively minor. The high frequency treatment maintains a relatively high level throughout the growth period. Compared with the low-frequency treatment, the yields of lint cotton and seed cotton in the high-frequency treatment have increased by 14.77% and 20.89%, respectively. Compared with the winter irrigation technology, there are no significant differences in the cotton yield and quality indicators of the "DSWE" high-frequency and high-seedling water treatment (W3D2). Over two years, the average unit yields of lint and seed cotton have decreased by 1.95% and 3.01%, respectively. Nevertheless, irrigation water during the growth period declined by 38.46%. The appropriate "DSWE" irrigation technology (W3D2) can significantly enhance the physiological indicators of cotton, ensuring crop yield and quality while significantly reducing the amount of agricultural irrigation water.

## 1 Introduction

Water scarcity significantly hampers sustainable agricultural development in numerous arid and semi-arid regions globally (Saco et al., 2020; Wang et al., 2023). One typical example is southern Xinjiang, characterized by intense sunlight, aridity, and minimal rainfall, with severely scarce freshwater resources (Yang et al., 2020; Ding et al., 2023). It is, however, an essential high-quality cotton production base and a typical type of purely irrigated agricultural area in China. It consumes up to 95% of agricultural water, primarily used for winter irrigation to leach soil salts and spring irrigation to maintain soil moisture pre-sowing (Feike et al., 2017; Ma et al., 2020a). However, the backwardness of water regulation technology and water resource management mode in arid areas has led to severe wastage of irrigation water, and the water resources in the region are decreasing. Consequently, meeting the water demand of crops during the reproductive stage, amidst the competing needs for salt washing and suppression during winter and spring irrigation periods, has become increasingly challenging (Li and Deng, 2021; Yang et al., 2022). The exploration and adoption of novel water-saving irrigation technologies have emerged as a pivotal strategy to enhance water resource utilization in Northwest China’s arid regions and to ensure the sustainable development of the agricultural economy (Fan et al., 2020; Cao et al., 2020).

The water control technology of “dry sowing and wet emergence (DSWE)” of cotton refers to the cotton field before sowing is no longer winter or spring irrigation, direct land preparation after laying the film and drip irrigation belt, and then cotton seeding, to reach the appropriate emergence temperature through the membrane drip irrigation method of a small amount of drip, so that the soil moisture under the membrane to meet the requirements of the cotton seedling emergence. This technology is essential in alleviating regional water shortages, saving costs, and increasing cotton farmers’ income. Integrating film moisture retention with drip irrigation, soil moisture content, and temperature can be effectively elevated, creating a favorable growth environment for cotton. This technology plays a pivotal role in mitigating regional water scarcity, reducing costs, and increasing the income of cotton farmers (Chen et al., 2021; Ma et al., 2020b). Comparatively, double-film mulching demonstrates notable advantages over single-film mulching, significantly enhancing cotton’s resilience to adverse climatic conditions and leveraging benefits such as temperature regulation, moisture preservation, soil condensation inhibition, and pest and disease prevention (Ai et al., 2011). In regions like Xinjiang Shihezi and Aksu, the widespread adoption of cotton double-film mulching technology has yielded favorable outcomes. In contrast to single-film mulching, it has led to a 13.5% reduction in pest and disease incidence, an impressive seedling emergence rate of 84.2%, and a substantial increase of 46.5 kg per hectare in average yield (Wu and Liu, 2008).

Under-membrane drip irrigation technology is used in the “DSWE” water control approach to conserve water, retain moisture, and preserve heat. This technique efficiently uses water resources to reduce temperature loss and soil moisture evaporation, resulting in soil and water conditions favorable for cotton seedlings’ growth. Luo et al. (2016) observed a significant decrease in photosynthetically active radiation of cotton leaves with increasing irrigation water quantity. Concurrently, the total biomass and biomass of organs increased by 6.5%-9.22% and 0.54%-1.4%, respectively, displaying a positive correlation between irrigation water quantity and photosynthetic indexes. Liu et al. (2022) and Zhang et al. (2016) noted that appropriate water irrigation, particularly high-water irrigation, significantly enhanced crop leaf photosynthetic capacity, while insufficient water irrigation led to photodamage in cotton leaves, thereby adversely affecting crop photosynthetic capacity. Additionally, drip frequency alongside irrigation water quantity may influence crop photosynthesis and yield characteristics. Li et al. (2017) observed that compared to low-frequency irrigation, high-frequency irrigation notably increased cotton leaf area index and dry matter accumulation, with a corresponding rise in group photosynthetic potential, group net assimilation rate, and group leaf area index. However, contrary findings indicated a negative correlation between cotton leaf area index and population physiological indicators, suggesting a decrease in physiological indicators with increased cotton leaf area index. Mahmood et al. (2021) and Wang et al. (2019a) investigated the effects of irrigation practices and mulch cover on physiological indicators of cotton. They found that high irrigation water treatments with significantly better water retention and thermal insulation under double-film cover significantly increased the photosynthetic rate of the cotton crop. Similarly, Karademir et al. (2011) and Ma et al. (2024) observed that increasing water gradient positively influenced cotton leaf area index, dry matter accumulation, and cotton yield. Consequently, the complexity of cotton photosynthesis, population physiological indicators, and yield quality characteristics due to various irrigation water gradients, drip frequencies, and mulching techniques underscores the necessity of identifying optimal irrigation modes to ensure cotton growth, improve yield quality, and enhance water utilization.

Because “DSWE” new water-saving irrigation technology does not need winter or spring irrigation, only in the seedling period for appropriate water irrigation to ensure the average growth of cotton, the water control program in the seedling period is essential. It may cause the following problems: (1) Seedling water irrigation may not be able to ensure the average growth of cotton seedling period, reducing the cotton Crop physiological indicators, affecting cotton photosynthesis, and reducing cotton yield quality. Seedling water too large may lead to cotton root rot, seriously affecting the cotton yield. (2) If the drip frequency is low, in the seedling stage of a one-time water irrigation, soil moisture leakage to the deep seedling cotton due to the shallow root system not effectively using soil moisture, resulting in reduced water utilization. (3) In the southern border region, where the temperature is higher and the daily evaporation is more significant, a smaller amount of water at seedling emergence or a lower frequency of dripping may lead to faster evaporation of soil moisture after irrigation, which is unable to maintain the growth of subsequent crops. Therefore, we hypothesized that different irrigation frequencies and seedling emergence water volume of “DSWE” had significant effects on crop physiology and yield quality indexes and that increasing the frequency of dripping in the seedling period and the amount of water in the seedling emergence could significantly improve the physiological indexes of the crop population, photosynthesis indexes, and the size of the yield quality. In this regard, through the 2-year “DSWE” water control experiment, to study the dry sowing and wet out water control drip frequency and seedling water volume on cotton photosynthesis, group physiological indicators and yield quality size of the cotton, aiming to achieve the following objectives: (1) Research “DSWE” water control drip frequency and seedling water volume on crop photosynthesis, population physiological indicators and yield quality. (2) Explore the correlation and interaction between cotton physiological growth indexes, yield, and fiber quality under the water control mode of “DSWE.” (3) Optimize the best water control scheme of the new water-saving irrigation technology of “DSWE.”

## 2 Materials and methods

### 2.1 Overview of the test site

**Figure 1:**
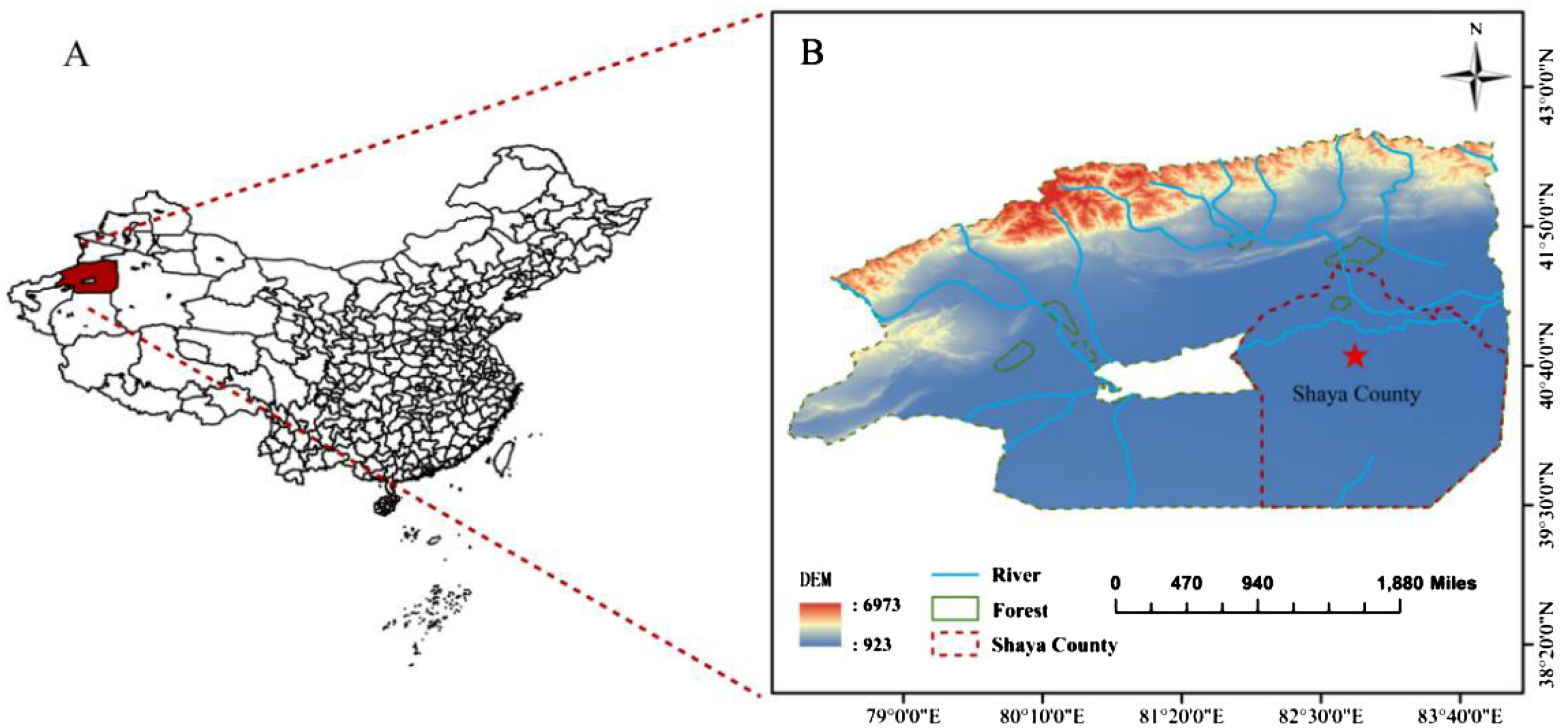
Location map of the study area. **(A)** is Aksu Region, Xinjiang, China; **(B)** is a Topographic map of Shaya County, Aksu Prefecture.

The experiment was conducted in Shaya County, Aksu Region, Xinjiang, Northwest China (latitude 41.22°N, longitude 82.78°E) (**Figure 1**). The study area exhibits a typical warm-temperate desert fringe climate, characterized by an average annual precipitation of merely 47.3 mm and a maximum annual evaporation of approximately 2000.7 mm, resulting in an evaporation-to-precipitation ratio of 42.3. Additionally, extreme weather phenomena such as wind, sandstorms, and hail are frequently influenced by neighboring desert sands and the Taklamakan Desert. Cotton stands as the primary cash crop within this locale. Soil physicochemical parameters in the study area are detailed in **Table 1**. The water table was approximately 3.8 m below the surface, and the soil pH was around 7.8.

**Table 1:** Physical and chemical properties of soil before irrigation.

Depth (cm)	Physical and chemical properties of soil before irrigation			
	Field water holding capacity (%)	Soil salt content, 2021 (g·kg^-1^)	Soil salt content, 2022 (g·kg^-1^)	Bulk density (g·cm^-3^)
0-20	25.42	1.79	1.84	1.47
20-40	24.96	2.35	1.69	1.48
40-60	29.94	1.22	3.27	1.62

### 2.2 Experimental design

From 2021 to 2022, three different seedling emergence water volumes were used, namely 6 mm (W1), 10.5 mm (W2), and 15 mm (W3), and two drip frequencies were designed, namely low frequency (2 times, D1) and high frequency (4 times, D2). Among them, the emergence water (W1, W2, W3) was completed at the emergence stage, and the remaining irrigation water was drip irrigation at the strong seedling stage. The water irrigation (emergence water) plus water irrigation at the strong seedling stage was the water irrigation quota at the seedling stage. The low frequency treatment (D1) was drip irrigation once at the emergence stage, and the strong seedling stage and high frequency treatment (D2) was drip irrigation twice at the emergence stage and the strong seedling stage (**Table 2**). At the same time, a local winter irrigation control treatment (CK) was designed, with an irrigation water volume of 225 mm. For CK winter irrigation treatment, the irrigation time was in November of the previous year, and due to the lower temperature than that in winter, frozen soil was formed after water irrigation to the soil, and it was thawed after the temperature rose in the following spring to ensure water consumption at the seedling stage. The irrigation effect of the soil surface was roughly the same as that of the treatment with a large amount of emerging water (W3), and the irrigation effect was better than that of the treatment with a low amount of emerging water (W1).

**Table 2:** Experimental design of the “DSWE” trial, 2021-2022.

Irrigation technology	Emergence water (mm)	Drip frequency (time)	Seedling irrigation quota (mm)			Treatment
			Emergence stage	Strong Seedling stage	Seedling stage	
“Dry sowing and wet emergence”	6.0 (W1)	2 (D1)	6.0	22.5	28.5	W1D1
		4 (D2)	4.5 + 1.5	10.5 + 12.0	28.5	W1D2
	10.5 (W2)	2 (D1)	10.5	22.5	33.0	W2D1
		4 (D2)	6.0 + 4.5	10.5 + 12.0	33.0	W2D2
	15.0 (W3)	2 (D1)	15.0	22.5	37.5	W3D1
		4 (D2)	10.5 + 4.5	10.5 + 12.0	37.5	W3D2
Winter irrigation	0	1	0	0	225.0	CK

**Figure 2:**
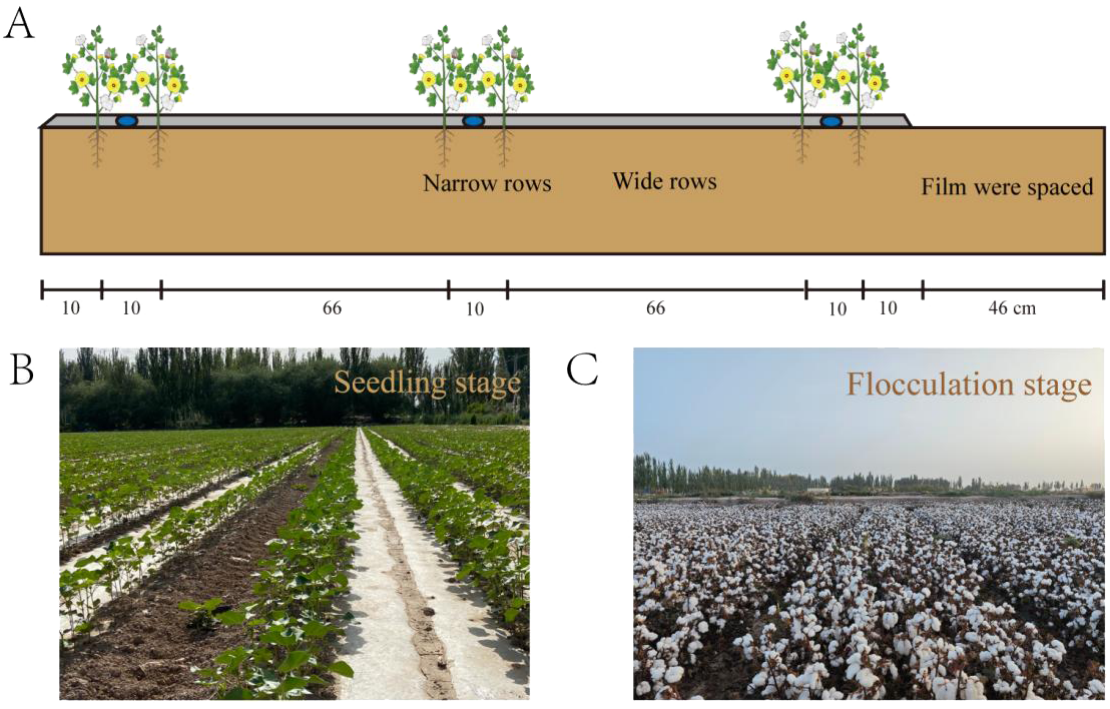
Planting pattern and drip irrigation belt layout in 2021-2022. **(A)** is a diagram of drip irrigation belt deployment for cotton, **(B)** is the growth of cotton at the seedling stage, and **(C)** is the growth of cotton at the flocculation stage.

**Figure 3:**
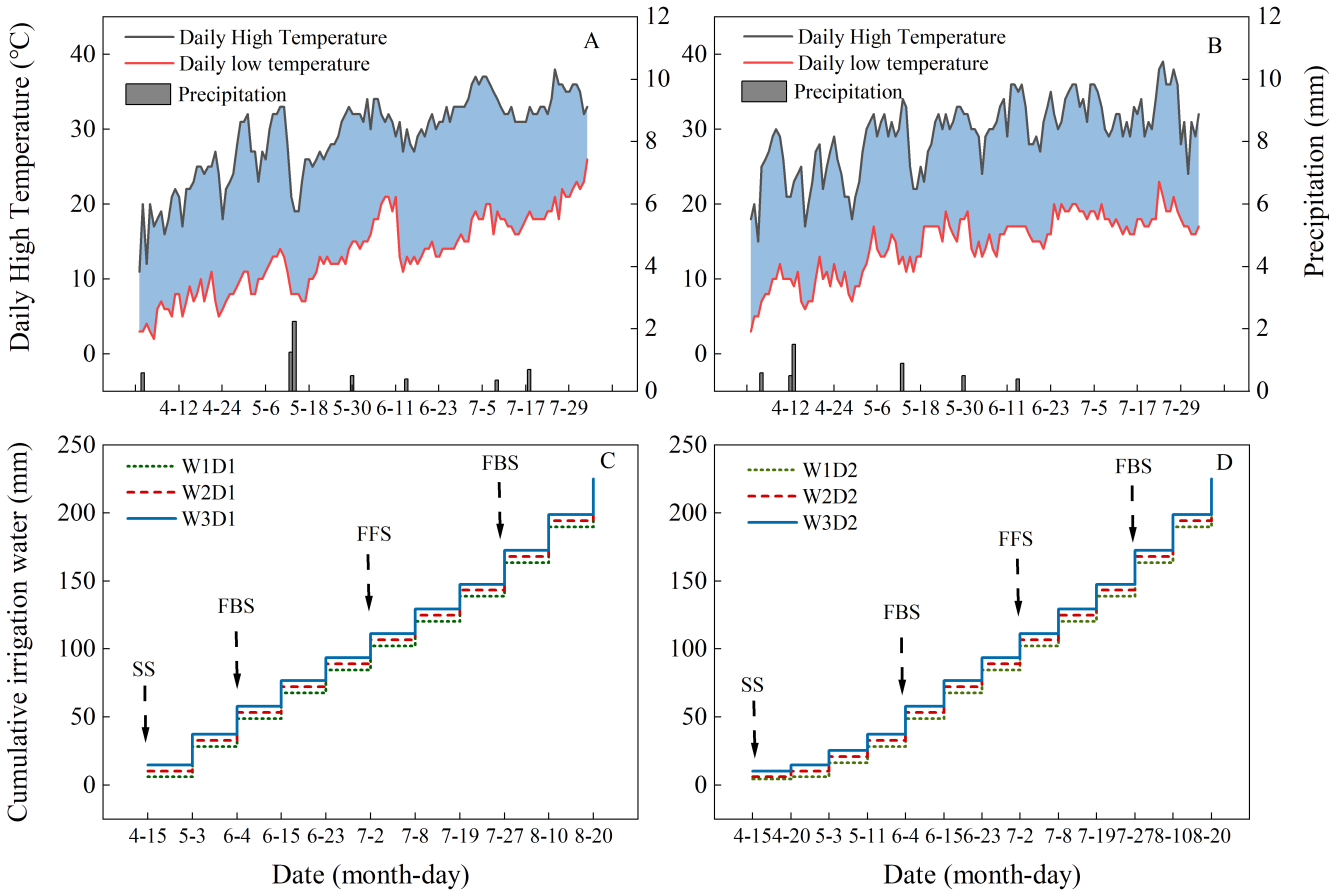
2021 and 2022 cotton growing season meteorological variations and irrigation schedules. **(A, B)** Show the daily variation of temperature and rainfall during the cotton growing season in 2021 and 2022, and **(C, D)** show the timing and amount of drip irrigation during the cotton growing season for the low-frequency (D1) and high-frequency (D2) treatments, respectively, for the years 2021-2022. SS, PBS, FFS, FBS, and FS denote the seedling, present bud, full flowering, full bolling, and flocculation stages of cotton, respectively.

The experiment conducted during 2021-2022 utilized the locally common cotton variety “Yuan Cotton No. 11” as the test crop, using a consistent planting configuration of 1 film, three tubes, and six rows. The experiment was conducted in a randomized split-plot design with three replicates in each plot, and the plot length was 10 m and width was 6 m. Fertiliser application and agronomic measures for cotton in the experimental site were based on local experience. The planting area was divided into wide rows, narrow rows, and bare ground between the film. Wide rows, narrow rows, and film were spaced at 66 cm, 10 cm, and 46 cm, respectively, with a plant spacing of 10 cm (**Figure 2**). Irrigation schedules and weather changes from 2021-2022 are shown in **Figure 3**, with water irrigated approximately every ten days from the present bud stage to the bell stage, with eight irrigations in 2021 and nine irrigations in 2022.

### 2.3 Indicators and methods

#### 2.3.1 Leaf area index determination

The LAI-2200C Plant Canopy Analyzer (LI-COR Biosciences, Inc, USA) was used to measure the leaf area index during the reproductive period of cotton in 2021-2022. The probe was placed horizontally above the cotton canopy for zeroing and then placed horizontally inside the cotton population. Different positions (wide, narrow, and bare ground between the membranes) were selected for measurements.

#### 2.3.2 Monitoring of relative chlorophyll values in cotton leaves

In 2021 and 2022, the relative chlorophyll value (SPAD) of the functional leaves of the main stems was determined using a SPAD-502 portable chlorophyll meter (Konica Minolta Holdings, Inc, JPN) during the reproductive period of cotton. The average value of the three parts of the leaves (the upper, middle, and lower parts) was calculated as the final SPAD value.

#### 2.3.3 Determination of photosynthesis parameters in cotton leaves

In 2021-2022, the photosynthetic performance of cotton plant leaves was determined at each reproductive period of cotton using a CI-340 handheld photosynthesis system (CID Bio-Science, Inc, USA) portable photosynthesis system tester, daily changes in photosynthesis were measured at 10:00, 12:00, 14:00, 16:00, 18:00 and 20:00 Beijing time. The natural light intensity was set before each measurement. The light intensity of the light source was set to be the same as that of the natural light intensity to minimize the influence of light intensity changes over time on the results. Then, the net photosynthetic rate (Pn), intercellular CO2 content (Ci), stomatal conductance (Gs), and transpiration rate (Tr) were measured in three cotton leaves under different treatments.

#### 2.3.4 Monitoring of physiological indicators in cotton populations

In 2021-2022, 10 cotton plants were successively selected in each plot to monitor the population’s physiological indexes during the reproductive period. The physiological indexes of each treatment include community photosynthetic potential (LAD) and population net assimilation rate (NAR) as below:


(1) $$LAD=\frac{2L_{2}+L_{1}}{2T_{2}-2T_{1}}$$

(2) $$NAR=\frac{M_{2}-M_{1}}{T_{2}-T_{1}}\times \frac{\text{ln}L_{2}-\text{ln}L_{1}}{L_{2}-L_{1}}$$

Where *L_1_* and *L_2_* (m^2^/hm^2^) are leaf area at *T_1_* and *T_2_* time, respectively; *M_1_* and *M_2_* (g/m^2^) are dry matter accumulation at *T_1_* and *T_2_*, respectively.

#### 2.3.5 Cotton production and composition factors

Three randomly selected plots measuring 2.0 m × 2.0 m were harvested by hand during the 2021 and 2022 cotton harvests. The harvested cotton was weighed and counted to determine the number of active bolls, boll weight, and cottonseed yield. The cotton was threshed using a threshing machine, and the threshed cotton was weighed to determine the lint yield. The harvest index (HI) was calculated by dividing the cotton yield by the dry matter.

#### 2.3.6 Cotton quality and component factors

At the harvest stage in 2021 and 2022, 50 randomly selected bolls from each plot were sent to the Cotton Quality Testing Centre of the Ministry of Agriculture (Urumqi, Xinjiang, China) for the determination of cotton fiber length, uniformity, strength, elongation, textile parameters, and staple fiber index. The fiber quality index (FQI) is determined below.


(3) $$\text{FQI}=\text{Fiber strength}\times \text{Length}\times \frac{\text{Uniformity}}{\text{Fineness}}$$

### 2.4 Data processing

The experiment was conducted in a completely randomized block design, with two drip frequencies (D1 and D2) and three seedling emergence volumes (W1, W2, and W3) in six treatments and three replications for each treatment. Two-factor analysis of variance (ANOVA) and multiple comparisons (*p*< 0.05) were performed using SPSS 25.0 to determine the differences in cotton physiological indices, yield, and quality between the treatments of emergence water volume and drip frequency. Principal Component Analysis (PCA) and correlation analysis of cotton yield and quality data using Origin2021 and plotting of correlation matrix. Using GeneCloud tools (https://www.genescloud.cn, March 30, 2024 access) on cotton physiological index interact ring heat map drawing; CAD2016 and ArcGIS 10.8 software were used to map the cotton planting pattern and the general situation of the experimental area.

## 3 Results

### 3.1 Cotton leaf area index

**Figure 4:**
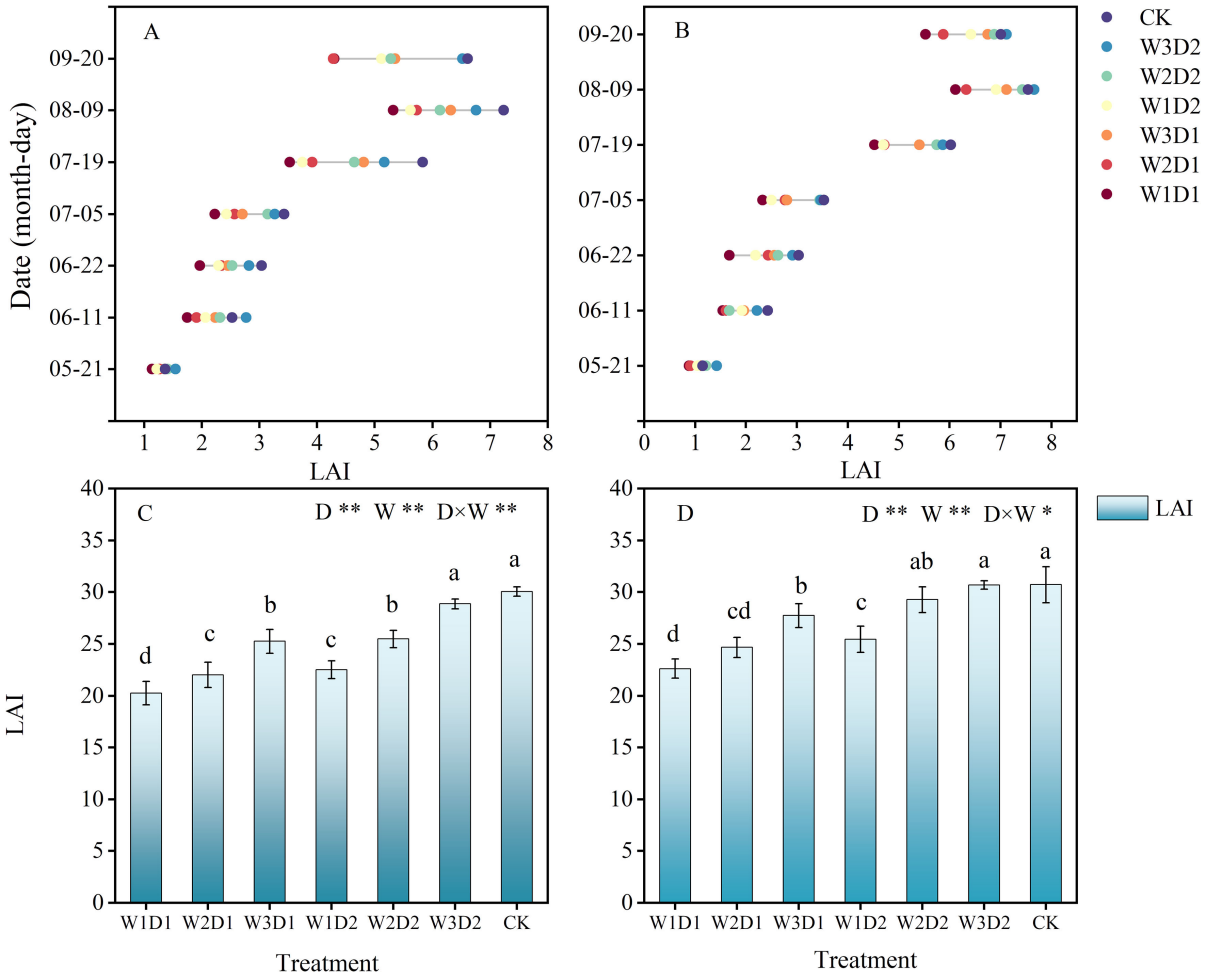
Changes of leaf area index in cotton growth period from 2021 to 2022. **(A, B)** Show the characteristics of LAI dynamics during the cotton growing season in 2021 and 2022, respectively; **(C, D)** show the analysis of variance of LAI histograms during the cotton growing season in 2021 and 2022, respectively; different lowercase letters indicate significant differences between treatments (*p*<0.05); D, drip frequency; W, emergence water, *p<0.05, **p<0.01.

From 2021 to 2022, the LAI of each treatment tended to increase and decrease with the advancing growth period, reaching a maximum at the bell stage (9th August) (**Figures 4A, B**). LAI has a consistent upward trajectory across treatments from the bud stage to the boll stage (21st May to 9th August). Notably, growth is slower during the transition from the bud stage to blooming (21st May to 5th July). Subsequently, during the boll stage (5th July to 9th August), LAI significantly increased, reaching its peak for the reproductive phase. However, leaf shedding occurs from the boll to the spathe stage (9th August to 20th September) due to insufficient water irrigation, initiating cotton spitting, and flocculation of its leaf area index.

The effects of drip frequency and emergence water on LAI were highly significant (*p*<0.01), and the interaction of drip frequency and emergence water volume (D×W) on LAI was substantial (*p*<0.05) between the two years (**Figures 4C, D**). When the frequency of dripping was the same, LAI showed a gradual increase with the increase of emergency water, and compared with the W1 treatment, LAI increased by 11.52% and 24.06% in W2 and W3 treatments, respectively. When the amount of emergence water was the same, the LAI of high frequency treatment (D2) was significantly more extensive than that of low frequency treatment (D1), and the LAI of D2 treatment increased by 13.84% compared with that of D1. In 2021-2022, the leaf area index of the CK treatment was not significantly different from that of the W3D2 treatment and was considerably more extensive than that of the remaining “DSWE” treatments.

### 3.2 Relative chlorophyll values for cotton

**Figure 5:**
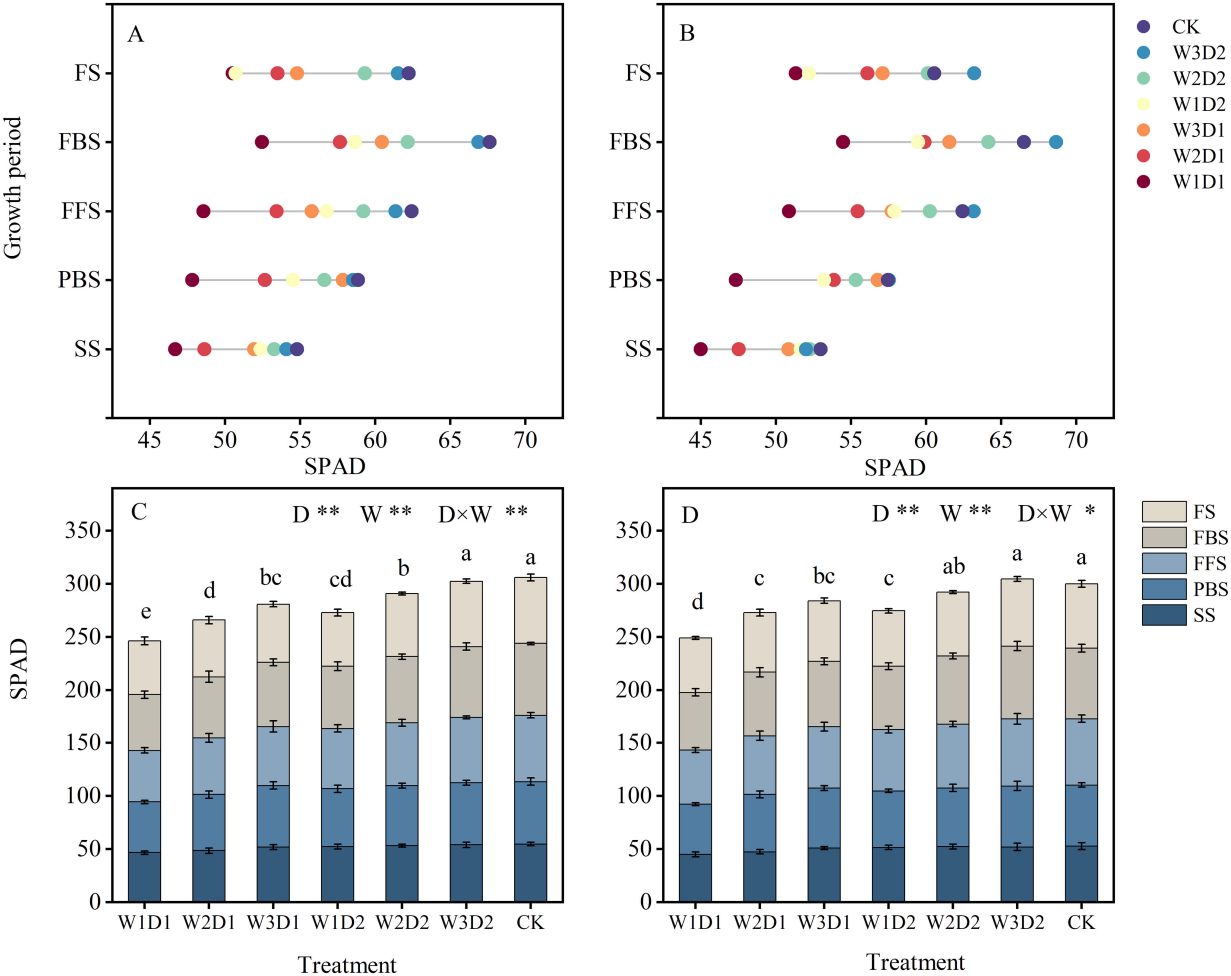
SPAD growth period changes in cotton leaves, 2021-2022. **(A, B)** Show the characteristics of SPAD dynamics during the cotton growing season in 2021 and 2022, respectively, and **(C, D)** show the analysis of variance of SPAD histograms during the cotton growing season in 2021 and 2022, respectively. Different lowercase letters indicate significant differences between treatments (*p*<0.05); D: drip frequency, W: emergence water, *: *p* <0.05, **: *p*<0.01.

The SPAD of each treatment tended to increase and decrease with the advancing growing period, reaching a maximum at the bell stage (**Figures 5A, B**). Both emergence water and drip frequency and their interactions(D×W) significantly affected SPAD in both years(*p*<0.05)(**Figures 5C, D**). When drip frequency was held constant, the performance of emergence water treatments in 2021-2022 followed the pattern W1< W2< W3, respectively. Similarly, when emergence water was consistent, SPAD values were significantly higher in high-frequency treatments compared to low-frequency treatments, with the sizes showing D1< D2 in 2021-2022 and no significant differences in SPAD values of W3D2 treatments compared with CK treatments. In comparison to the CK treatment, reductions in SPAD values were observed for the W1D1, W2D1, W3D1, W1D2, W2D2, and W3D2 treatments in 2021 by 14.78%, 11.26%, 5.20%, 4.43%, 2.79%, and 1.28%, respectively. Similarly, in 2022, the W1D1, W2D1, W3D1, W1D2, W2D2, and W3D2 treatments showed decreases of 15.09%, 10.32%, 4.09%, 2.51%, 1.38%, and 1.89%, respectively.

### 3.3 Cotton leaf photosynthesis

#### 3.3.1 Daily changes in leaf photosynthesis during bloom

**Figure 6:**
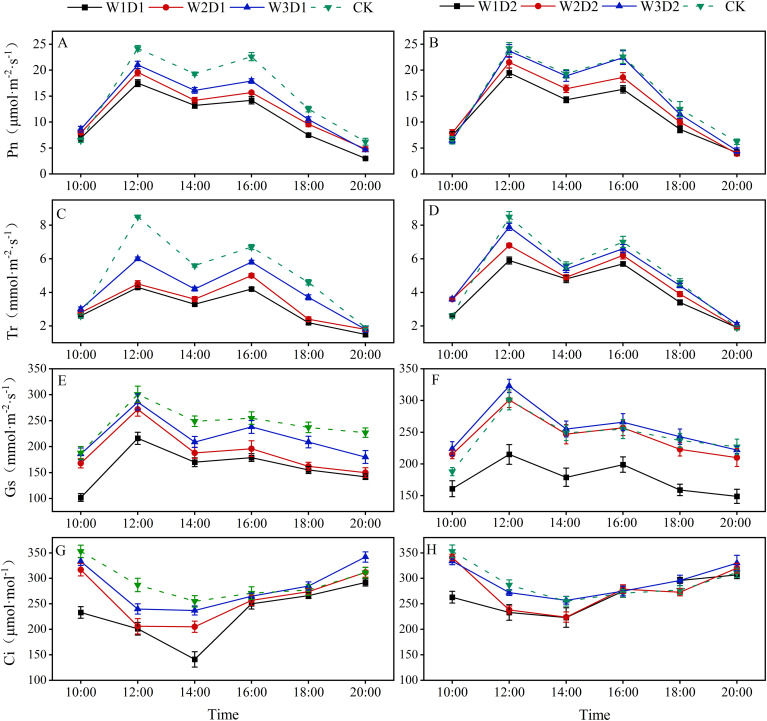
Diurnal variation of photosynthetic indices. **(A, C, E, G)** respectively show the daily changes of Ci, Gs, Tr, and Pn under the low frequency treatment (D1) of “DWSE.” **(B, D, F, H)** respectively show the daily changes of Ci, Gs, Tr, Pn under high frequency treatment (D2) of “DWSE”.

The daily changes in net photosynthetic rate (Pn), transpiration rate (Tr), and stomatal conductance (Gs) across different water control treatments exhibited a consistent pattern characterized by a double-peak curve (**Figure 6**). Conversely, the fluctuations in carbon dioxide concentration (Ci) followed a single-peak curve, decreasing initially before rising again. In each “DSWE” treatment, the peaks for Pn, Tr, and Gs occurred around 12:00 and 16:00. In contrast, Ci peaked around 14:00. Between 12:00 and 14:00, as sunlight intensified with rising temperatures, water transpiration in cotton leaves decreased, leading to automatic closure of stomata and a gradual reduction in Gs, accompanied by weakened leaf cell respiration and a decrease in Ci. Light intensity reached a maximum at 14:00, and Pn showed some reduction. From 14:00 to 16:00, as sunlight waned and temperatures decreased, stomata in the treatment leaves gradually reopened, resulting in a slight rebound in stomatal conductance and a gradual increase in Ci values, eventually reaching their peak. Overall, it is observable that the photosynthesis indices within one day for each “DSWE” low frequency treatment (D1) were significantly lower than those of the CK treatment. Among the high frequency treatments, there was no significant difference in the photosynthesis indices of Pn, Tr, and Gs between the W3D2 and CK treatments. In contrast, the Gs photosynthesis index was significantly larger.

#### 3.3.2 Changes in leaf photosynthesis during cotton growing period

**Figure 7:**
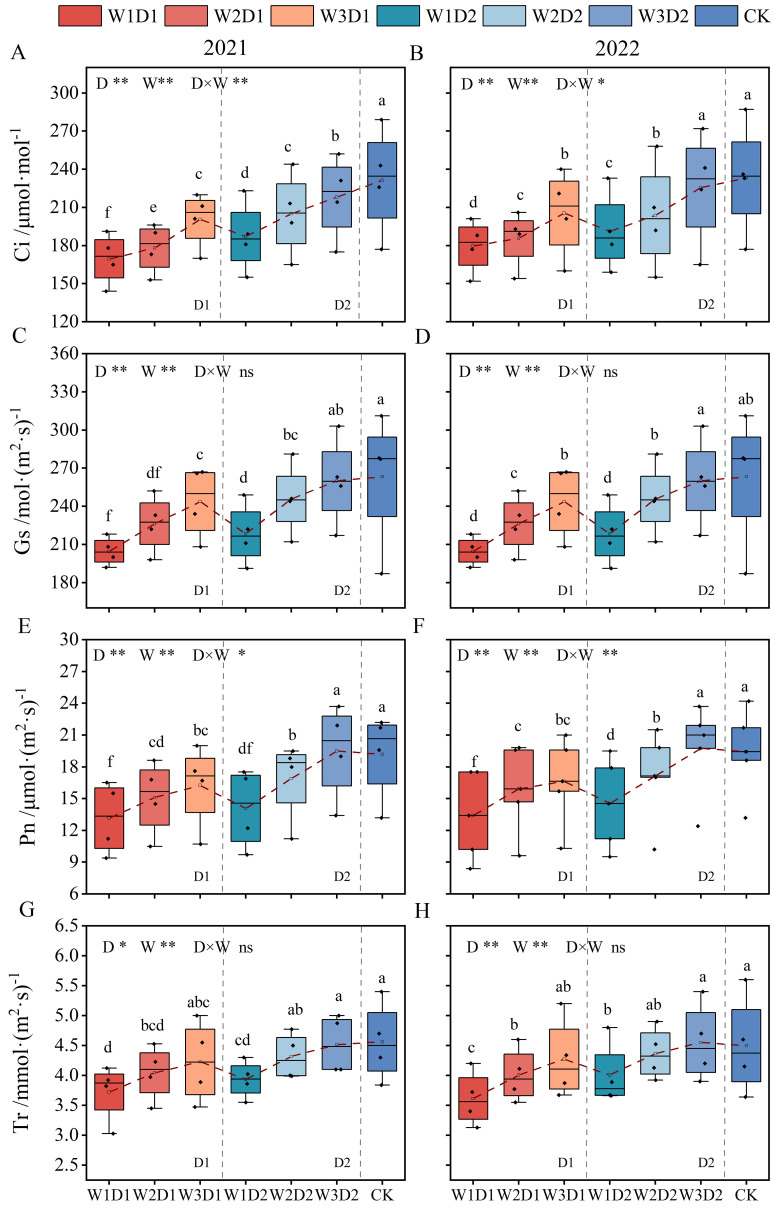
Effect of emergence water and drip frequency on photosynthesis indexes of cotton during growing period in 2021 and 2022. **(A, C, E, G)** the significance analyses of Ci, Gs, Pn, Tr in 2021 and **(B, D, F, H)** show Ci, Gs, Pn, Tr in 2022, respectively. *p<0.05, **p<0.01, ns: *p*>0.05. Different lowercase letters indicate significant differences between treatments (*p*<0.05).

In both years, the effects of drip frequency and emergence water on cotton leaf Ci and Gs were highly significant (*p*<0.01) (**Figures 7A–D**). The drip frequency and emergence water interaction significantly affected Ci (p<0.05) but not Gs. With the increase of emergence water, the Ci and Gs values of cotton leaves showed a gradually increasing trend, which was W1<W2<W3 in 2021-2022. Ci and Gs were significantly more significant in the high-frequency treatment with the same emergence water, increasing by 10.03% and 6.67%, respectively, compared with the low frequency treatment. Significantly more minor differences in Ci and Gs were observed in the W3D2 treatment compared to the CK treatment, with Ci decreasing by 4.53% and Gs increasing by 0.13% over the two years.

In both years, drip frequency and emergence water significantly (*p*<0.05) affected cotton leaf Pn and Tr, and the interaction of both drip frequency and emergence water significantly (*p*<0.05) affected Pn, but not Tr (**Figures 7E–H**). With increased water emergence, cotton leaf Pn and Tr values gradually rose. Compared to W1, W2 and W3 leaf Pn increased by 17.87% and 25.90%, respectively, and W2 and W3 leaf Tr increased by 9.39% and 13.02%, respectively. When the emergence water remained constant, Pn and Tr values of high-frequency treatments significantly exceeded those of low frequency treatments; Pn and Tr increased by 12.22% and 7.60%, respectively, in two years. There was no significant difference in Pn and Tr in the W3D2 treatment compared to the CK treatment, which increased by 1.68% and 0.12%, respectively, over the two years. As a whole, there was no significant difference in photosynthesis indexes of W3D2 treatment during the growth period under the dry sowing and wet extraction water irrigation mode compared with CK winter irrigation, indicating that the appropriate dry sowing and wet extraction irrigation mode could improve the photosynthesis indexes of crop growth period and ensure the formation of crop yield and quality.

### 3.4 Physiological indicators of the cotton population

**Figure 8:**
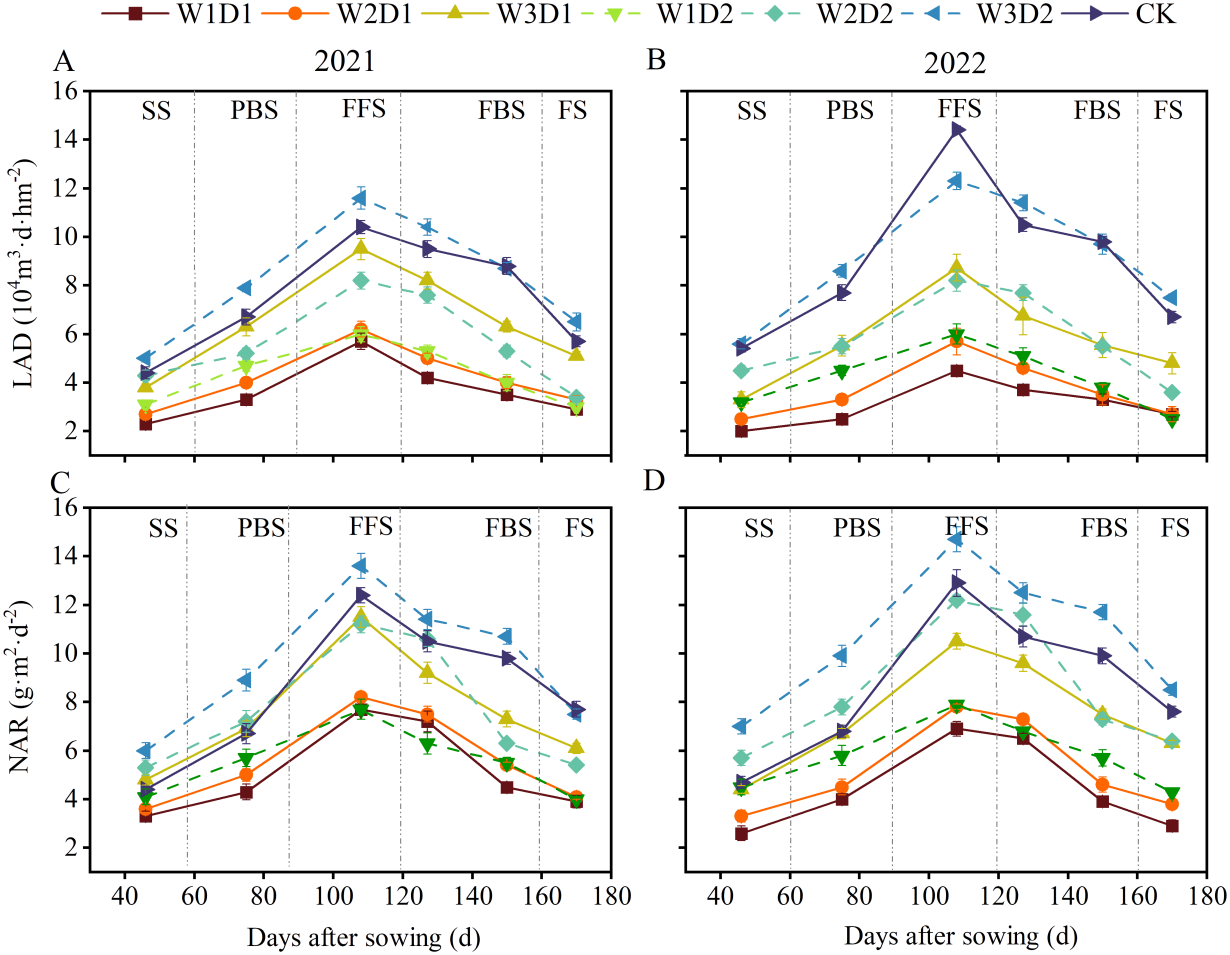
Changes of physiological indexes of cotton population in 2021-2022. **(A, B)** Shows the LAD growth period changes in 2021 and 2022, respectively, and **(C, D)** shows the NAR growth period changes in 2021 and 2022, respectively. SS, PBS, FFS, FBS, and FS denote the seedling, present bud, full flowering, full bolling, and flocculation stages of cotton, respectively.

During the two years, the population physiological indexes of each treatment tended to increase and decrease as the cotton growth period progressed, and the population physiological indexes peaked at 108 days after sowing (FFS) (**Figure 8**). When the drip frequency is the same, the differences between the different emergence water treatments are minor in the seedling and bud stages. Then the differences are gradually significant in the boll stage (108d after sowing), and then the differences decrease in the flocculating stage (170d after sowing); when the emergence water is the same, the high frequency treatment (D2) maintains a more significant level throughout the growth cycle.

**Figure 9:**
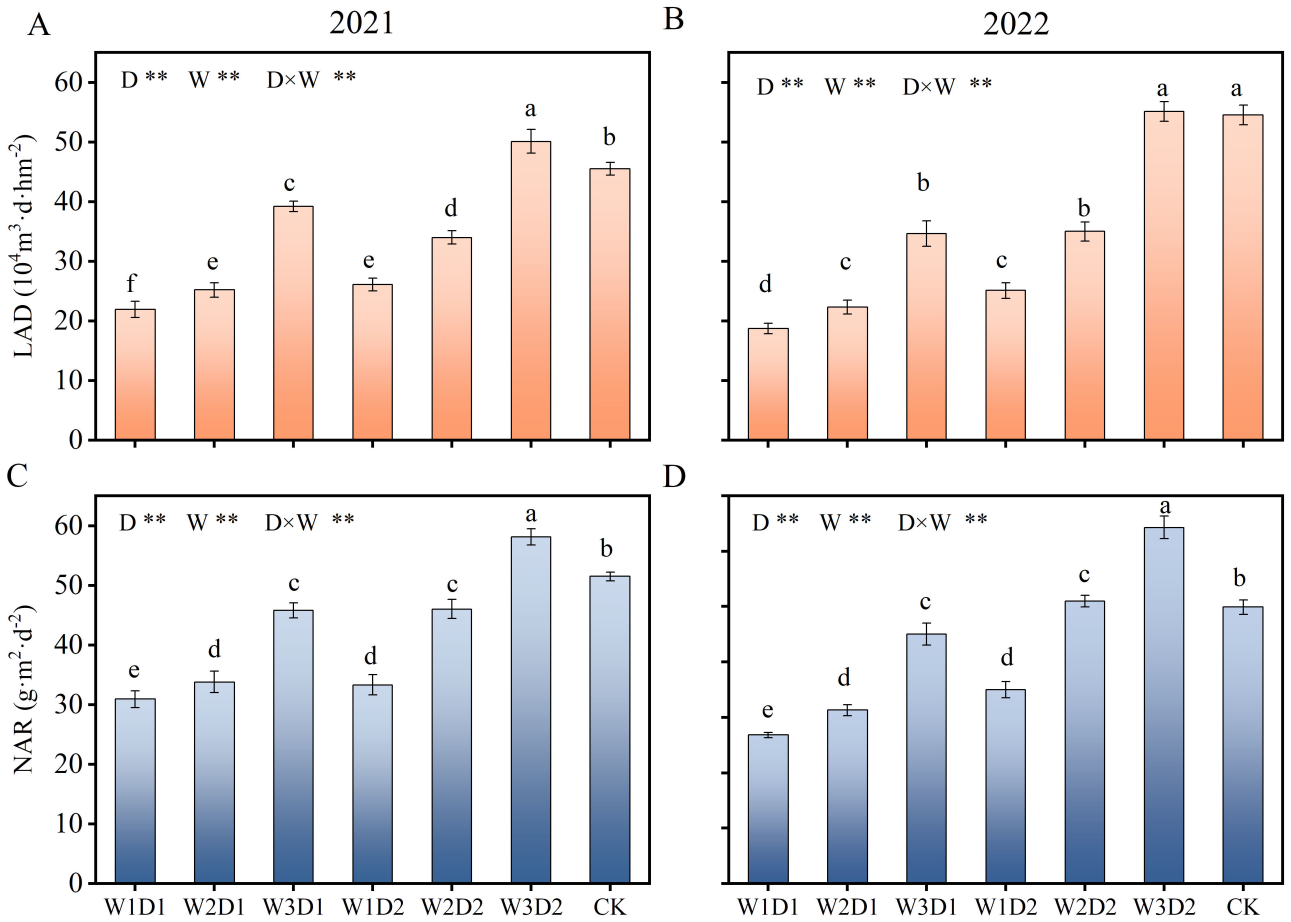
Effect of drip frequency and emergence water volume on physiological indicators of cotton population, 2021-2022. **(A, B)** Show the significance analysis of LAD in 2021 and 2022, respectively, and **(C, D)** show the significance analysis of NAR in 2021 and 2022, respectively. Different lowercase letters indicate significant differences between treatments (*p*<0.05); D: drip frequency, W: emergence water, *: *p* <0.05, **: *p*<0.01.

Seedling emergence water, drip frequency, and the interaction between the two (D×W) had highly significant (p<0.01) effects on LAD and NAR (**Figure 9**). At the same drip frequency, NAR and LAD roughly showed a gradual increase with increasing emergence water, and compared with W1, LAD increased by 26.01% and 93.94% in W2 and W3 treatments, respectively, and NAR increased by 27.51% and 68.58% in W2 and W3 treatments, respectively. At the same amount of emergency water, LAD and NAR were significantly more significant in the high-frequency therapies than in the low frequency treatments. They increased by 38.68% and 34.52% in the D2 treatment LAD and NAR, respectively, compared with D1. Compared with CK treatment, W3D2 treatment LAD and NAR were significantly less different; LAD and NAR increased by 3.49% and 19.06%, respectively.

### 3.5 Cotton yield and fiber quality indicators

#### 3.5.1 Cotton yield and harvest indices

Drip frequency and emergence water significantly influenced seed cotton and lint yield (*p*< 0.01). However, drip frequency and emergence water did not exhibit a significant impact on the HI (*p* > 0.05) (**Table 3**). When drip frequency was constant, increasing emergence water gradually increased lint and seed cotton yields. Similarly, when the emergence of water remained constant, high-frequency treatments resulted in significantly higher yields than low frequency treatments. Specifically, in 2021 and 2022, high-frequency treatments led to an increase of 15.89% and 13.64% in lint yield and 23.60% and 18.17% in seed cotton yield compared to low-frequency treatments. Among the “DSWE” water management treatments, the W3D2 treatments with high frequency and sizeable seedling emergence volume exhibited the highest yields. Compared to the CK treatment, in 2021, lint and seed cotton yields decreased by 4.33% and 3.84%, respectively, while in 2022, lint yield increased by 0.43%, and seed cotton yield decreased by 2.18%. Irrigation water usage was reduced by 31.45% and 45.47% in 2021 and 2022, respectively. With the increasing emergence of water, the number of bolls per plant and boll weight gradually increased. Furthermore, high-frequency treatments demonstrated significantly higher values than low-frequency treatments. In 2021, there were no significant differences in the harvest index, while in 2022, high-frequency treatments exhibited slightly higher harvest index values than low-frequency treatments.

**Table 3:** Cotton production indicators for 2021-2022.

Year	Treatment	Irrigation quota (m^3^·hm^-2^)	Yield Indicators					
			Bells (pc)	Bell weight (g)	Dry matter accumulation (DMA)	Lint production (kg·ha^-1^)	Seed cotton production (kg·ha^-1^)	Harvest index (HI)
2021	W1D1	2983	3.50b	3.72b	16799.27f	2110.4e	4252.3e	0.253a
	W2D1	3208	3.41b	3.82b	17374.19e	2315.6d	4527.5d	0.260a
	W3D1	3433	4.00a	4.21a	19628.79d	2477.3c	4876.3c	0.248a
	W1D2	2983	3.82b	4.31c	20377.94d	2223.4d	4473.7d	0.220b
	W2D2	3208	4.15b	4.55b	21215.19c	2775.6b	5301.5b	0.250a
	W3D2	3433	4.25a	5.15a	22756.68b	3001.5a	5743.3a	0.252a
	CK	5008	4.11a	5.21a	23949.18a	3121.3a	6003.5a	0.264a
	D	–	*	*	**	**	**	ns
	W	–	ns	*	**	**	**	ns
	D*W	–	ns	ns	**	**	**	ns
2022	W1D1	2158.35	4.80b	4.92b	13944.50f	1898.5d	4004.68c	0.287a
	W2D1	2203.35	4.81b	4.92b	16907.90e	1969.6d	4243.06b	0.251b
	W3D1	2248.35	5.10a	5.41a	18287.35d	2513.4b	5216.08a	0.285a
	W1D2	2158.35	4.92b	5.11c	19844.55c	2211.3c	4382.16c	0.221c
	W2D2	2203.35	5.05b	5.65b	22958.90b	2663.6b	5255.86b	0.229c
	W3D2	2248.35	5.35a	6.05a	23878.75a	3012.5a	6272.82a	0.263b
	CK	4123.35	5.41a	6.11a	23918.65a	3079.5a	6246.22a	0.261b
	D	–	*	*	**	**	**	*
	W	–	ns	ns	**	**	**	ns
	D*W	–	ns	ns	**	**	**	ns

Different lowercase letters indicate significant differences between treatments (*p*<0.05); D, drip frequency; W, emergence water, *p<0.05, **p<0.01, ns: *p*>0.05.

#### 3.5.2 Cotton quality and component factors

drip frequency and emergence water significantly affected cotton fiber length, uniformity, textile parameters, and Fiber Quality Index (FQI) (*p*< 0.05). At the same time, they had no significant impact on fiber strength, elongation, and maturity (*p* > 0.05) (**Table 4**). The interaction between drip frequency and emergency water did not significantly affect fiber quality. When drip frequency was held constant, increasing emergence water generally led to a gradual increase in fiber length, uniformity, and textile parameters. Across 2021-2022, the treatments exhibited a pattern where W1D1< W2D1< W3D1, W1D2< W2D2< W3D2. Similarly, when emergence water was held constant, high-frequency treatments showed significantly better fiber quality indicators than low frequency treatments. Specifically, in 2021, cotton fiber length, uniformity, and textile parameters increased by 2.18%, 2.89%, and 4.83%, respectively, while in 2022, they increased by 3.48%, 1.95%, and 10%, respectively, in high-frequency treatments compared to low-frequency treatments. The FQI in the W3D2 treatments was significantly higher than in the other “DSWE” treatments and did not differ considerably from the CK treatment. Compared to the CK treatment, the FQI decreased by 7.71% in 2021 and 14.3% in 2022 in the W3D2 treatments.

**Table 4:** Cotton quality indicators in 2021-2022.

Year	Treatment	Cotton quality indicators						
		Fiber strength (cN/tex)	Fiber Length (mm)	Uniformity (%)	Elongation rate (%)	Maturity	Textile parameters	FQI
2021	W1D1	28.13b	29.96b	81.7c	10.7a	0.84a	125d	19126.31d
	W2D1	28.46b	29.46b	82.8c	10.6a	0.83a	132c	19283.93d
	W3D1	28.77b	31.33a	84.5b	10.8a	0.84a	136b	21157.02c
	W1D2	29.45b	30.98b	84.3b	10.5a	0.82a	135b	21364.45c
	W2D2	29.66b	30.57b	85.4b	10.7a	0.83a	138b	21509.09c
	W3D2	30.13b	31.18a	86.5a	10.6a	0.82a	139b	22572.98b
	CK	32.44a	31.02a	87.5a	9.4b	0.85a	149a	24458.41a
	D	ns	*	*	ns	ns	*	*
	W	ns	*	*	ns	ns	*	*
	D*W	ns	ns	ns	ns	ns	ns	ns
2022	W1D1	27.6b	27.96b	80.9c	10.0a	0.83a	121c	17341.72f
	W2D1	27.7b	28.46b	81.9c	10.6a	0.82a	119c	17934.78e
	W3D1	28.0b	29.33a	83.1b	10.9a	0.82a	130b	18956.96d
	W1D2	29.1b	28.98c	83.2b	10.4a	0.83a	136b	19490.02c
	W2D2	29.3b	29.57b	83.6b	10.8a	0.82a	137b	20119.76b
	W3D2	28.9b	30.18a	83.9b	10.7a	0.82a	134b	20327.15b
	CK	33.0a	30.02b	86.2a	9.8b	0.84a	151a	23720.80a
	D	ns	*	*	ns	ns	*	*
	W	ns	*	*	ns	ns	*	*
	D*W	ns	ns	ns	ns	ns	ns	ns

Different lowercase letters indicate significant differences between treatments (*p*<0.05); D, drip frequency; W, emergence water, *p<0.05, ns: *p*>0.05.

#### 3.5.3 Principal component analysis of cotton yield and quality components

**Figure 10:**
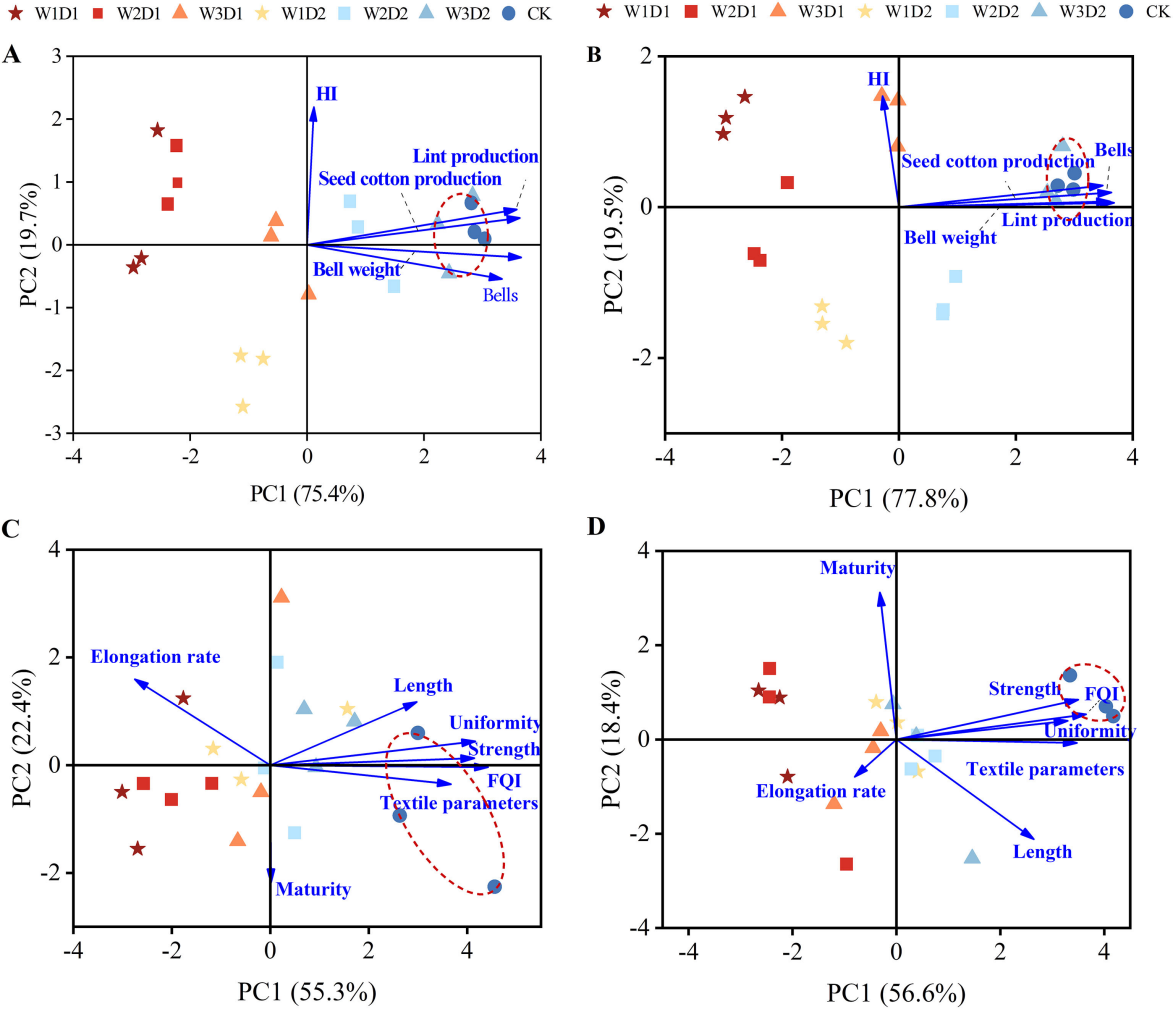
Principal component analysis map of cotton yield and quality from 2021 to 2022. **(A, B)** Show the principal component analysis of yield indicators in 2021-2022, **(C, D)** show the principal component analysis of quality indicators in 2021 and 2022.

Principal Component Analysis (PCA) was conducted on the five yields and seven quality indicators of each “DSWE” water control treatment and the winter irrigation control treatment. The combined loadings plot and scores plot are depicted in **Figure 10**. Analysis of the yield composition from 2021 to 2022 (**Figures 10A, B**) revealed that the first two principal components had relatively large eigenvalues and cumulative variance contribution rates, with eigenvalues greater than 1, indicating a comprehensive reflection of all sample information. In 2021, Principal Component 1 and Principal Component 2 contained 75.4% and 19.7% of the original information, respectively, while in 2022, they contained 77.8% and 19.5% of the original information, respectively. Analyzing the angle between the loading directions and the projection distance of each yield indicator in **Figures 10A, B** for 2021-2022 uncovered positive correlations among all yield indicators. Notably, boll weight significantly influenced lint and seed cotton yields. Examination of the treatment scores in **Figures 10A, B** revealed a high similarity in yield indicators between the W3D2 and CK groups, while the remaining water control treatments showed low similarity. Furthermore, the projection magnitude along the PC1 yield principal component direction in **Figures 10A, B** demonstrated that the variance of the projection of the winter irrigation control treatment (CK) and the high-frequency, high-emergence water volume combination (W3D2) along the PC1 yield principal component direction was significantly greater than that of the other “DSWE” treatments. This indicates that CK and W3D2 carry considerably more information in each yield indicator, leading to higher comprehensive evaluation indicators obtained from PCA dimensionality reduction analysis.

In Figure, axes 1 and 2 respectively accounted for 55.3% and 22.4% of the total variance in cotton quality composition for 2021, while in **Figure 10D**, axes 1 and 2 respectively accounted for 56.6% and 18.4% of the total variance in cotton quality composition for 2021. Examination of the angular direction and projection distance of each quality indicator in the figures revealed a significant negative correlation between the elongation rate and the Fiber Quality Index (FQI) and significant negative correlations between other indicators and FQI. Notably, fiber length and uniformity exerted a more substantial influence on FQI. Analysis of the scores for each treatment in **Figures 10C, D** indicated that the CK treatment showed relatively low similarity with the quality indicators of each “DSWE” treatment. In contrast, the similarity among quality indicators of the “DSWE” treatments was notably higher. Furthermore, examination of the projection magnitude along the PC1 direction for each quality indicator in **Figures 10C, D** demonstrated that the variance of the projection of the CK treatment along the PC1 quality principal component direction was significantly greater than that of each “DSWE” treatment. Among high-frequency therapies, there was relatively minor variability in projection variance. These findings suggest that the winter irrigation control treatment carries significantly more information in each quality indicator, leading to higher comprehensive evaluation indicators obtained from PCA dimensionality reduction analysis.

#### 3.5.4 Correlation analysis of cotton yield and quality with physiological parameters

**Figure 11:**
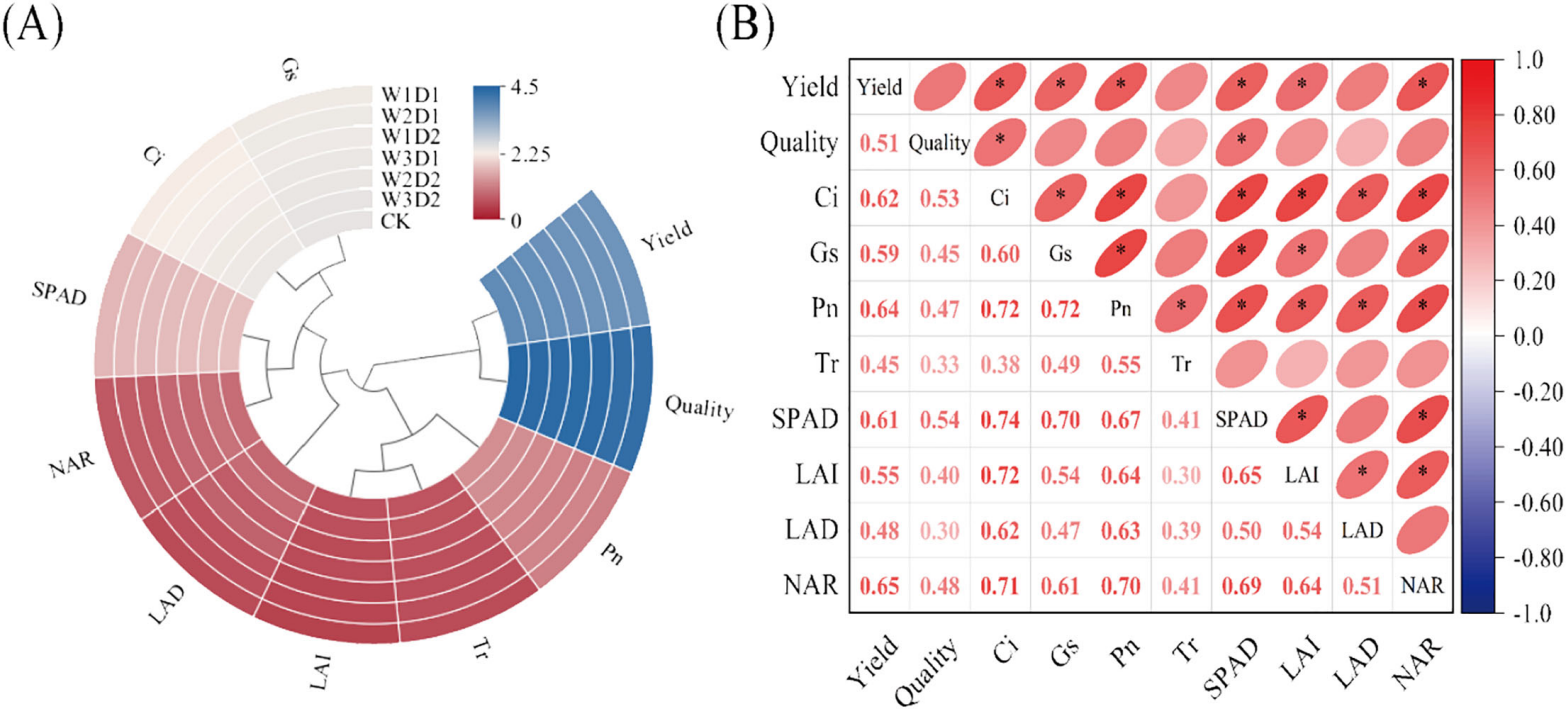
Cotton physiological indicators interaction ring heat map and correlation matrix, **(A)** is the heat map of cotton physiological indicators, the color close to blue indicates that the heat value of the indicator is higher, and the color close to red indicates that the heat value of the indicator is lower; **(B)** is the correlation matrix of cotton physiological indicators, the darker the color shows that the correlation between the indicators is higher, the red color indicates that the indicators are positively correlated with each other, the blue color indicates that the indicators are negatively correlated with each other, and the * indicates that the indicators are significantly associated with each other (*p<* 0.05).

To assess the correlation between physiological indices under “DSWE” water control and cotton yield and quality, an interactive circular heatmap and correlation matrix (**Figure 11**) were generated. The heatmap demonstrates that the 12 variables are roughly organized into three primary clusters: Yield and quality constitute one cluster with notably higher heat values; LAI, Tr, and Pn form another cluster with the lowest heat values; and Gs, Ci, SPAD, and NAR roughly constitute a cluster with heat values in the middle range. Correlation analysis indicates that Ci, Pn, Gs, SPAD, and NAR are closely associated with Yield, with NAR displaying the highest correlation coefficient (0.65). Quality is closely linked to Ci and SPAD, with SPAD exhibiting a notably higher correlation coefficient with quality indicators (0.54). Overall, it is apparent that photosynthetic indices and population physiological indices collectively contribute to determining the magnitude of cotton yield and quality indicators.

## 4 Discussion

### 4.1 Cotton photosynthetic characteristics

Photosynthesis is the fundamental driving force for plant growth and constitutes the basis for biomass accumulation and yield formation (Evans, 2013). A higher canopy photosynthetic rate provides a solid material foundation for plant growth and development (Terashima and Hikosaka, 1995). This study found that within the appropriate range of “DSWE” water control, treatments with higher post-emergence watering levels promote cotton photosynthetic indices. With the same developmental stage factor, cotton leaf Pn and Tr values generally showed an increasing trend with increasing emergence water. Compared to W1, W2 and W3 leaf Pn increased by 17.87 and 25.90%, respectively, and W2 and W3 leaf Tr increased by 9.39 and 13.02%, respectively. Zou et al. (2022) found that insufficient irrigation water might be the main reason for the significant decrease in net photosynthetic rate, stomatal conductance, and transpiration rate of cotton leaves. Chastain et al. (2014) study confirmed this, showing that compared with the high-water treatment, the low-water treatment significantly inhibited photosynthesis, with markedly lower net photosynthetic rate, transpiration rate, and stomatal conductance indices. However, Wang et al. (2019b) argued that the primary reason might be the synergistic effect of stomatal and non-stomatal variables leading to a decline in cotton leaf photosynthetic rate, with mild water stress having a minor impact on photosynthesis. It has also been shown that stomata close during mild to moderate water stress, leading to a decrease in intercellular CO_2_ concentration, resulting in a reduced photosynthetic rate. Prolonged water stress can damage photosynthetic organs, thereby reducing the net photosynthetic rate of plants (Zahid et al., 2021).

This study found that the photosynthetic characteristics of cotton under “DSWE” high-frequency treatments were significantly higher than those under low-frequency treatments. Ci and Gs were substantially larger in the high-frequency therapy with the same emergence water, increasing by 10.03% and 6.67%, respectively, compared with the low-frequency treatment. This is consistent with the findings of Liang et al. (2021), which related to photosynthetic characteristics research. Low-frequency treatments decreased net photosynthetic rate due to non-stomatal limiting factors, resulting in significantly lower maximum photochemical efficiency, photochemical quenching coefficient, and photochemical quantum yield compared to high-frequency treatments. The variation in soil water content during the growth period may contribute to the size of photosynthetic indices (Loka et al., 2011). Water significantly affects crop growth and development at each stage. Throughout the cotton growth process, especially during the blooming and boll formation stages, cotton plants exhibit vigorous growth and reproductive development, resulting in a significant increase in water demand (Sahito et al., 2015). All water is applied at once with low frequency treatments, resulting in water stress as the growth period progresses. Water stress leads to a decrease in stomatal conductance, increased respiration, obstruction of photosynthetic product transport, and reduced photosynthetic function period, thereby impairing the plant’s ability to maintain efficient photosynthetic production capacity throughout the entire growth period (Chen et al., 2022; Wu et al., 2023). Insufficient water may affect the cotton net photosynthetic rate, stomatal conductance, and other photosynthetic indices. Only appropriate “DSWE” post-emergence watering amounts and drip frequency (W3D2) can facilitate cotton leaf photosynthesis, providing a solid foundation for cotton growth and development.

### 4.2 Cotton leaf area index and population physiological indexes

The leaf area index is one of the most important parameters determining the accurate calculation of the rate of canopy photosynthesis and is an essential indicator of the quality of the crop population (Pauli et al., 2017; Bunce, 1989). Relevant studies have found minimal variation in LAI among different water control treatments during the seedling period. As the growth period progresses, the LAI of each treatment gradually increases, reaching its maximum value during the blooming and boll formation stages, and the differences among treatments also become increasingly significant (Li et al., 2020; Cetin et al., 2023), which is consistent with the LAI-related research in this experiment. Through variance analysis, this study found that within the appropriate range of “DSWE” water control, the LAI of cotton significantly increases with the increase in the emergence of water, and high-frequency water control can effectively enhance the LAI of cotton. LAI increased by 11.52% and 24.06% in W2 and W3 treatments, respectively. When the amount of emergence water was the same, the LAI of high frequency treatment (D2) was significantly more extensive than that of low frequency treatment (D1), and the LAI of D2 treatment increased by 13.84% compared with that of D1. Some scholars have found that increasing irrigation quotas and frequencies can promote the nutritional growth of cotton and enhance the LAI during the budding and blooming stages (Kalaydjieva et al., 2015). Therefore, besides irrigation water quantity and drip frequency, factors such as planting density and mulching methods may also need to be considered when assessing the LAI of cotton.

Zhao et al. (2016) have shown that the physiological indicators of the cotton population are significantly higher with larger irrigation quotas, which are more conducive to cotton dry matter accumulation and ultimately increasing yields. Consistent with these findings, our experiment observed that the physiological indicators of the cotton population gradually increased with the increase in post-emergence watering. Among different drip frequency treatments, those with higher frequencies exhibited more considerable physiological indicators. Compared with W1, LAD increased by 26.01% and 93.94% in W2 and W3 treatments, respectively, and NAR increased by 27.51% and 68.58% in W2 and W3 treatments, respectively. Increased by 38.68% and 34.52% in the D2 treatment LAD and NAR, respectively, compared with D1. The differences observed in physiological indicators among treatments in our experiment may be related to cotton emergence rate and leaf area index. The emergence rate was lower in treatments with higher irrigation quotas, affecting the cotton population structure. However, the cotton canopy received richer natural resources, such as better ventilation and light in these treatments, promoting early photosynthesis and dry matter production (Feng et al., 2013). In contrast, differences in emergence rates among treatments with different drip frequencies were relatively small, making the soil moisture environment in the main root zone a determining factor for cotton population physiological indicators (Ballester et al., 2021).

### 4.3 Cotton yield and quality indexes

In this study, the yield of cotton seed cotton lint increased gradually with the increase of seedling emergence water, and the yield of high frequency treatment was significantly larger. Compared with low frequency treatment (D1), the yield of high frequency treatment (D2) lint and seed cotton increased by 15.89%, 13.64%, 23.60%, and 18.17%, respectively, in 2021 and 2022. This is consistent with the findings of Ballester et al. (2021) and Hill et al. (2024) regarding the effect of different irrigation frequencies on cotton yield. Cotton boll number and yield varied under different water stress conditions. Mild water stress during the entire growth period had a minimal impact on cotton yield, while continuous water stress significantly reduced the number of bolls per plant. Boll formation concentrated on cotton plants’ lower and middle fruiting branches, resulting in fewer bolls on the upper branches, leading to decreased cotton yield (Cheng et al., 2021; He et al., 2022). Consistent with these findings, our experiment suggests that the reduction in post-emergence watering may concentrate the main nutrient and reproductive growth of cotton plants on the lower and middle parts, where the root system and lower fruiting branches and leaves grow vigorously. Conversely, the upper fruiting branches and leaves receive less water irrigation, affecting the net photosynthetic intensity and leading to a decrease in non-structural carbohydrate content, exacerbating the shedding of cotton bolls and consequently reducing yield.

The results showed that drip frequency and emergence water had significant effects on cotton fiber length, evenness, textile parameters, and FQI but had no significant effects on fiber strength, elongation, and maturity. As post-emergence watering and drip frequency increased, the average length of the upper half of the fiber, fiber uniformity, textile parameters, and Fiber Quality Index (FQI) all increased to varying degrees. The differences in fiber strength, elongation, and maturity among the various “DSWE” water control treatments were relatively small. However, findings by Dai et al. (2022) and Gao et al. (2021) differed; they found that reduced water irrigation could increase cotton fiber uniformity and elongation, thus improving cotton quality. Studies by Thorp et al. (2020) found that the decrease in irrigation volume had an increasing impact on the average length of the upper half of cotton fibers. This was mainly manifested as the aggravation of water stress with decreasing irrigation volume, leading to a significant decrease in cotton fiber quality. Furthermore, it was shown that water stress at any stage resulted in reduced fiber uniformity in cotton. Therefore, cotton quality can be influenced by different irrigation methods, cultivation practices, growing environments, and cotton varieties.

## 5 Conclusion

Compared with traditional winter and spring irrigation, “DSWE” irrigation technology can greatly reduce the water consumption of agricultural irrigation under the condition of guaranteeing crop yield and quality, which is crucial for the sustainable development of agriculture in arid areas. The results of the study showed that, under the “DSWE” irrigation mode, high frequency treatment could significantly improve crop physiological indexes, compared with low frequency treatment (D1), high frequency treatment (D2) of cotton Ci, Gs increased by 10.03% and 6.67%, respectively, with the increase in the amount of emergence of cotton population physiological indexes increased significantly, compared with the W1, the W3 treatment of the LAD and NAR increased by 93.94% and 68.58%, respectively. Compared with CK, there was no significant difference in yield and quality indicators in W3D2 treatment, and lint and seed cotton yields decreased by 1.95% and 3.01% on average in two years. Still, irrigation water use decreased by 38.46% in the growing season. After PCA dimensionality reduction analysis, CK and W3D2 treatments carried significantly more information in each yield index, and the comprehensive evaluation indexes were substantially higher, but in the analysis of quality indexes, CK treatment was significantly larger than each “DSWE” treatment. Therefore, the W3D2 “DSWE” irrigation scheme was recommended as a sustainable production strategy for cotton fields in the arid region of Xinjiang, China, to improve water use efficiency and reduce agricultural irrigation water use. However, the “DSWE” irrigation management is adaptive and highly influenced by site-specific changes in the climatic environment, which should be considered in further research.

## Data Availability

The original contributions presented in the study are included in the article/**Supplementary Material**. Further inquiries can be directed to the corresponding author.
