# Erratum: Response of photosynthesis, population physiological indexes, and yield of cotton in dry areas to the new technology of “dry sowing and wet emergence”

**DOI:** 10.3389/fpls.2024.1528014

**Published:** 2024-11-26

**Authors:** Frontiers Production Office

**Affiliations:** Frontiers Media SA, Lausanne, Switzerland

**Keywords:** arid region, photosynthesis, population physiological index, quality, water management, yield

Due to a production error, there was a mistake in affiliation 1. Instead of “Xinjiang Institute of Water Resources and Hydropower Research, Drought and Water Hazard Defence Institute, Urumqi, China”, it should be “College of Water Resources, North China University of Water Resources and Electric Power, Zhengzhou, China”.

Due to a production error, there was a mistake in affiliation 2. Instead of “Xinjiang Institute of Water Resources and Hydropower Research, Urumqi, China”, it should be “Xinjiang Institute of Water Resources and Hydropower Research, Drought and Water Hazard Defence Institute, Urumqi, China”.

The publisher apologizes for this mistake.

The original version of this article has been updated.

