# Supplementary material for: Response of photosynthesis, population physiological indexes, and yield of cotton in dry areas to the new technology of “dry sowing and wet emergence”
Source: Front Plant Sci. 2024 Oct 17;15:1487832. doi: 10.3389/fpls.2024.1487832 (PMC11526576; doi:10.3389/fpls.2024.1487832)
Supplement: Supplementary file 1 [file Table1.docx]

Supplementary Material

# Supplementary Data

**Supplementary material （Data support for the graphs in the chapters）:**

# 3 Results

## 3.1 Cotton leaf area index

**Fig. 4.** Changes of leaf area index in cotton growth period from 2021 to 2022.

| Year | Date | W1D1 | W2D1 | W3D1 | W1D2 | W2D2 | W3D2 | CK |
| --- | --- | --- | --- | --- | --- | --- | --- | --- |
| 2021 | 05-21 | 1.14 | 1.27 | 1.36 | 1.22 | 1.41 | 1.55 | 1.37 |
|  | 06-11 | 1.75 | 1.91 | 2.24 | 2.07 | 2.32 | 2.77 | 2.53 |
|  | 06-22 | 1.97 | 2.34 | 2.46 | 2.29 | 2.53 | 2.82 | 3.04 |
|  | 07-05 | 2.23 | 2.57 | 2.71 | 2.43 | 3.15 | 3.27 | 3.43 |
|  | 07-19 | 3.53 | 3.92 | 4.81 | 3.75 | 4.65 | 5.17 | 5.83 |
|  | 08-09 | 5.32 | 5.73 | 6.32 | 5.62 | 6.13 | 6.76 | 7.24 |
|  | 09-20 | 4.3 | 4.28 | 5.35 | 5.12 | 5.28 | 6.52 | 6.61 |
| 2022 | 05-21 | 0.88 | 0.91 | 1.12 | 1.05 | 1.21 | 1.43 | 1.15 |
|  | 06-11 | 1.55 | 1.61 | 1.96 | 1.92 | 1.67 | 2.22 | 2.43 |
|  | 06-22 | 1.67 | 2.44 | 2.56 | 2.19 | 2.63 | 2.92 | 3.04 |
|  | 07-05 | 2.33 | 2.77 | 2.81 | 2.5 | 3.45 | 3.47 | 3.53 |
|  | 07-19 | 4.53 | 4.72 | 5.41 | 4.7 | 5.75 | 5.87 | 6.03 |
|  | 08-09 | 6.12 | 6.33 | 7.12 | 6.92 | 7.43 | 7.66 | 7.54 |
|  | 09-20 | 5.53 | 5.88 | 6.75 | 6.42 | 6.88 | 7.12 | 7.01 |

## 3.2 Relative chlorophyll values for cotton

**Fig. 5.** SPAD growth period changes in cotton leaves, 2021-2022.

| Year | Growing period | W1D1 | W2D1 | W3D1 | W1D2 | W2D2 | W3D2 | CK |
| --- | --- | --- | --- | --- | --- | --- | --- | --- |
| 2021 | SS | 46.7 | 48.63 | 51.95 | 52.37 | 53.27 | 54.1 | 54.8 |
|  | PBS | 47.83 | 52.66 | 57.87 | 54.53 | 56.63 | 58.53 | 58.87 |
|  | FFS | 48.56 | 53.44 | 55.77 | 56.79 | 59.22 | 61.37 | 62.43 |
|  | FBS | 52.47 | 57.66 | 60.44 | 58.67 | 62.18 | 66.87 | 67.63 |
|  | FS | 50.53 | 53.52 | 54.81 | 50.72 | 59.33 | 61.52 | 62.24 |
| 2022 | SS | 45 | 47.53 | 50.83 | 51.67 | 52.27 | 52 | 53 |
|  | PBS | 47.33 | 53.87 | 56.77 | 53.23 | 55.33 | 57.53 | 57.47 |
|  | FFS | 50.87 | 55.47 | 57.73 | 57.9 | 60.27 | 63.17 | 62.43 |
|  | FBS | 54.47 | 59.9 | 61.57 | 59.47 | 64.17 | 68.67 | 66.53 |
|  | FS | 51.33 | 56.12 | 57.11 | 52.22 | 60.13 | 63.22 | 60.54 |

## 3.3 Cotton leaf photosynthesis

### 3.3.1 Daily changes in leaf photosynthesis during bloom

**Fig. 6.** Diurnal variation of photosynthetic indices.

| Photosy  -nthetic index | Time | W1D1 | W2D1 | W3D1 | W1D2 | W2D2 | W3D2 | CK |
| --- | --- | --- | --- | --- | --- | --- | --- | --- |
| Ci | 10:00 | 233 | 317 | 333 | 263 | 341 | 334 | 353 |
|  | 12:00 | 201 | 206 | 240 | 233 | 238 | 272 | 287 |
|  | 14:00 | 141 | 205 | 237 | 223 | 224 | 257 | 255 |
|  | 16:00 | 250 | 257 | 265 | 275 | 279 | 275 | 271 |
|  | 18:00 | 266 | 274 | 285 | 296 | 273 | 296 | 277 |
| Gs | 10:00 | 102 | 168 | 186 | 161 | 215 | 224 | 188 |
|  | 12:00 | 216 | 272 | 286 | 215 | 301 | 323 | 301 |
|  | 14:00 | 170 | 188 | 209 | 179 | 247 | 255 | 249 |
|  | 16:00 | 179 | 196 | 238 | 199 | 257 | 266 | 255 |
|  | 18:00 | 155 | 162 | 209 | 159 | 223 | 243 | 237 |
| Pn | 10:00 | 6.9 | 7.9 | 8.6 | 7.4 | 7.9 | 6.5 | 6.5 |
|  | 12:00 | 17.5 | 19.6 | 21 | 19.5 | 21.5 | 23.7 | 24.2 |
|  | 14:00 | 13.2 | 14.2 | 16.1 | 14.3 | 16.4 | 18.9 | 19.3 |
|  | 16:00 | 14.2 | 15.7 | 17.9 | 16.3 | 18.6 | 22.4 | 22.6 |
|  | 18:00 | 7.5 | 9.6 | 10.5 | 8.6 | 10 | 11.5 | 12.5 |
| Tr | 10:00 | 2.6 | 2.8 | 3 | 2.6 | 3.6 | 3.6 | 2.6 |
|  | 12:00 | 4.2 | 4.6 | 5.2 | 4.8 | 4.9 | 5.4 | 5.6 |
|  | 14:00 | 4.3 | 4.5 | 6 | 5.9 | 6.8 | 7.9 | 8.5 |
|  | 16:00 | 3.3 | 5 | 6 | 5.7 | 6.2 | 6.6 | 7 |
|  | 18:00 | 2.2 | 2.4 | 3.7 | 3.4 | 3.9 | 4.4 | 4.6 |

### 3.3.2 Changes in leaf photosynthesis during cotton growing period

**Fig. 7.** Changes in photosynthesis during cotton growing period.

| Photosy  -nthetic index | Growing period | 2021 | | | | | | | 2022 | | | | | | |
| --- | --- | --- | --- | --- | --- | --- | --- | --- | --- | --- | --- | --- | --- | --- | --- |
|  |  | W1D1 | W2D1 | W3D1 | W1D2 | W2D2 | W3D2 | CK | W1D1 | W2D1 | W3D1 | W1D2 | W2D2 | W3D2 | CK |
| Ci | SS | 144 | 4.13 | 153 | 2.55 | 170 | 3.56 | 155 | 2.44 | 165 | 3.22 | 175 | 5.21 | 177 | 4.33 |
|  | PBS | 178 | 4.17 | 190 | 4.75 | 211 | 4.23 | 189 | 3.09 | 198 | 4.5 | 214 | 4.33 | 226 | 3.04 |
|  | FFS | 191 | 3.56 | 196 | 5.72 | 220 | 5.63 | 223 | 4.44 | 244 | 3.04 | 252 | 5.25 | 279 | 5.05 |
|  | FBS | 165 | 2.25 | 173 | 5.1 | 201 | 3.22 | 181 | 5.01 | 213 | 4.55 | 231 | 6.52 | 243 | 5.09 |
| Gs | SS | 192 | 198 | 208 | 191 | 212 | 217 | 187 | 192 | 198 | 208 | 191 | 212 | 217 | 187 |
|  | PBS | 208 | 233 | 267 | 222 | 246 | 263 | 278 | 208 | 233 | 267 | 222 | 246 | 263 | 278 |
|  | FFS | 218 | 252 | 266 | 249 | 281 | 303 | 311 | 218 | 252 | 266 | 249 | 281 | 303 | 311 |
|  | FBS | 200 | 222 | 234 | 211 | 244 | 256 | 277 | 200 | 222 | 234 | 211 | 244 | 256 | 277 |
| Pn | SS | 9.4 | 10.5 | 10.7 | 9.7 | 11.2 | 13.4 | 13.2 | 8.4 | 9.6 | 10.3 | 9.5 | 10.2 | 12.4 | 13.2 |
|  | PBS | 11.2 | 14.5 | 16.7 | 12.2 | 18 | 21.9 | 19.6 | 10.2 | 14.7 | 15.7 | 11.2 | 17 | 21.9 | 18.6 |
|  | FFS | 16.5 | 18.6 | 20 | 17.5 | 19.5 | 23.7 | 22.2 | 17.5 | 19.6 | 21 | 19.5 | 21.5 | 23.7 | 24.2 |
|  | FBS | 15.5 | 16.8 | 17.6 | 16.9 | 18.8 | 19 | 21.7 | 17.5 | 19.8 | 19.6 | 17.9 | 19.8 | 21 | 21.7 |
| Tr | SS | 3.82 | 3.97 | 3.89 | 3.86 | 3.99 | 4.1 | 3.84 | 3.4 | 3.77 | 3.87 | 3.66 | 3.92 | 3.9 | 3.64 |
|  | PBS | 3.92 | 4.23 | 4.55 | 4.02 | 4.77 | 4.87 | 4.7 | 3.72 | 4.11 | 4.34 | 3.89 | 4.52 | 4.7 | 4.6 |
|  | FFS | 4.12 | 4.53 | 5 | 4.3 | 4.5 | 5 | 5.4 | 4.2 | 4.6 | 5.2 | 4.8 | 4.9 | 5.4 | 5.6 |
|  | FBS | 3.03 | 3.45 | 3.47 | 3.55 | 4 | 4.1 | 4.3 | 3.13 | 3.55 | 3.67 | 3.67 | 4.13 | 4.2 | 4.15 |

## 3.4 Physiological indicators of cotton population

**Fig. 8.** Changes of physiological indexes of cotton population in 2021-2022.

| Population physiological indicators | Days after sowing/d | 2021 | | | | | | | 2022 | | | | | | |
| --- | --- | --- | --- | --- | --- | --- | --- | --- | --- | --- | --- | --- | --- | --- | --- |
|  |  | W1D1 | W2D1 | W3D1 | W1D2 | W2D2 | W3D2 | CK | W1D1 | W2D1 | W3D1 | W1D2 | W2D2 | W3D2 | CK |
| LAD | 46 | 2.3 | 2.7 | 3.8 | 3.1 | 4.3 | 5 | 4.4 | 2 | 2.5 | 3.3 | 3.2 | 4.5 | 5.6 | 5.4 |
|  | 75 | 3.3 | 4 | 6.3 | 4.7 | 5.2 | 7.9 | 6.7 | 2.5 | 3.3 | 5.52 | 4.5 | 5.5 | 8.6 | 7.7 |
|  | 108 | 5.7 | 6.2 | 9.5 | 6 | 8.2 | 11.6 | 10.4 | 4.5 | 5.7 | 8.73 | 6 | 8.2 | 12.3 | 14.4 |
|  | 127 | 4.2 | 5 | 8.2 | 5.3 | 7.6 | 10.4 | 9.5 | 3.7 | 4.6 | 6.75 | 5.1 | 7.7 | 11.4 | 10.5 |
|  | 150 | 3.5 | 4 | 6.3 | 4 | 5.3 | 8.7 | 8.8 | 3.3 | 3.5 | 5.55 | 3.8 | 5.5 | 9.7 | 9.8 |
|  | 170 | 2.9 | 3.3 | 5.1 | 3 | 3.4 | 6.5 | 5.7 | 2.7 | 2.7 | 4.8 | 2.5 | 3.6 | 7.5 | 6.7 |
| NAR | 46 | 3.3 | 3.6 | 4.8 | 4.1 | 5.3 | 6 | 6.7 | 4 | 4.5 | 6.7 | 5.8 | 7.8 | 9.9 | 6.8 |
|  | 75 | 4.3 | 5 | 6.9 | 5.7 | 7.2 | 8.9 | 12.4 | 6.9 | 7.8 | 10.5 | 7.9 | 12.2 | 14.7 | 12.9 |
|  | 108 | 7.7 | 8.2 | 11.5 | 7.7 | 11.2 | 13.6 | 10.5 | 6.5 | 7.3 | 9.6 | 6.8 | 11.6 | 12.5 | 10.7 |
|  | 127 | 7.2 | 7.5 | 9.2 | 6.3 | 10.6 | 11.4 | 9.8 | 3.9 | 4.6 | 7.5 | 5.7 | 7.3 | 11.7 | 9.9 |
|  | 150 | 4.5 | 5.4 | 7.3 | 5.5 | 6.3 | 10.7 | 7.7 | 2.9 | 3.8 | 6.3 | 4.3 | 6.4 | 8.5 | 7.6 |
|  | 170 | 3.9 | 4.1 | 6.1 | 4 | 5.4 | 7.5 | 6.7 | 4 | 4.5 | 6.7 | 5.8 | 7.8 | 9.9 | 6.8 |

**
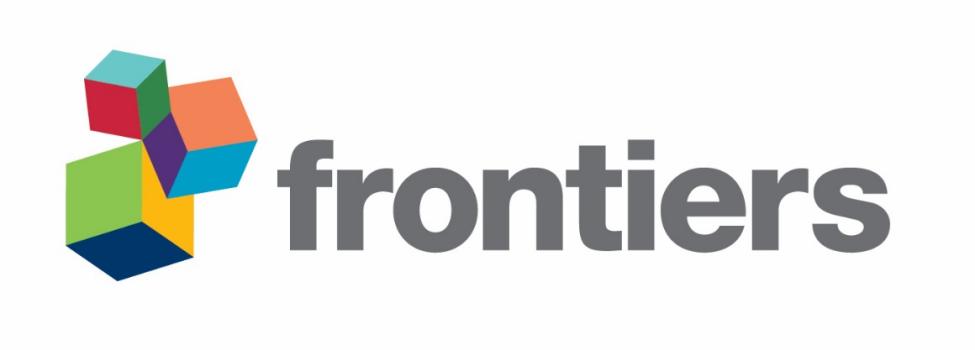
**

**Supplementary Figure 1.** The figure legends are required to have the same font as the main text, 12 point normal Times New Roman, single spaced. Please use a single paragraph for each legend and prepare the figures keeping in mind the PDF layout.
